# Differential Impact of Subtherapeutic Antibiotics and Ionophores on Intestinal Microbiota of Broilers

**DOI:** 10.3390/microorganisms7090282

**Published:** 2019-08-22

**Authors:** Kelsy Robinson, Sage Becker, Yingping Xiao, Wentao Lyu, Qing Yang, Huiling Zhu, Hua Yang, Jiangchao Zhao, Guolong Zhang

**Affiliations:** 1 Department of Animal and Food Sciences, Oklahoma State University, Stillwater, OK 74078, USA; 2 Institute of Quality and Standards for Agro-Products, Zhejiang Academy of Agricultural Sciences, Hangzhou 310021, China; 3 Hubei Key Laboratory of Animal Nutrition and Feed Science, Wuhan Polytechnic University, Wuhan 430000, China; 4 Department of Animal Science, Division of Agriculture, University of Arkansas, Fayetteville, AR 72701, USA

**Keywords:** microbiota, antibiotics, ionophores, antimicrobial growth promoters, chickens

## Abstract

Antimicrobial growth promoters (AGPs) are commonly used in the livestock industry at subtherapeutic levels to improve production efficiency, which is achieved mainly through modulation of the intestinal microbiota. However, how different classes of AGPs, particularly ionophores, regulate the gut microbiota remains unclear. In this study, male Cobb broiler chickens were supplemented for 14 days with or without one of five commonly used AGPs including three classical antibiotics (bacitracin methylene disalicylate, tylosin, and virginiamycin) and two ionophores (monensin and salinomycin) that differ in antimicrobial spectrum and mechanisms. Deep sequencing of the V3-V4 region of the bacterial 16S rRNA gene revealed that two ionophores drastically reduced a number of rare bacteria resulting in a significant decrease in richness and a concomitant increase in evenness of the cecal microbiota, whereas three antibiotics had no obvious impact. Although each AGP modulated the gut microbiota differently, the closer the antibacterial spectrum of AGPs, the more similarly the microbiota was regulated. Importantly, all AGPs had a strong tendency to enrich butyrate- and lactic acid-producing bacteria, while reducing bile salt hydrolase-producing bacteria, suggestive of enhanced metabolism and utilization of dietary carbohydrates and lipids and improved energy harvest, which may collectively be responsible for the growth-promoting effect of AGPs.

## 1. Introduction

Subtherapeutic antimicrobial growth promoters (AGPs) are commonly included in livestock diets to improve production performance [1,2]. This is particularly true in the poultry industry where supplementation has been shown to improve weight gain and feed efficiency, inhibit pathogen growth, and reduce mortality [2,3]. However, increased microbial resistance linked to antibiotic use in food animals has led to a ban on AGPs in both the European Union and the U.S.A. and a change in consumer preference towards antibiotic-free production [1,4,5]. This has, therefore, created a need to develop antibiotic alternatives to ensure animal health and growth performance.

While the exact mode of action remains elusive, AGPs are postulated to provide performance benefits through modulation of the intestinal microbiota [2,6,7,8]. Indeed, the inability of antibiotics to improve growth performance in germ-free chicks has provided compelling evidence that antibiotics work primarily by reshaping the intestinal microbiota [9], which is a unique ecosystem known to play a vital role in host health and metabolism through its effects on feed digestion, nutrient absorption, vitamin synthesis, and immune system development [10,11,12]. Consistently, a link between the intestinal microbiota and growth performance of livestock animals has been established [7,13,14]. For example, two specific fecal bacterial community structures known as enterotypes are significantly associated with body weight and average daily gain of pigs [15]. Similar studies in broiler chickens have revealed a strong correlation between certain intestinal bacterial taxa and weight gain [16]. Specific AGP-induced changes in the intestinal microbiota are beginning to be elucidated [2,7,8]. However, the wide range of AGPs used between studies, as well as differences in animal age, diet, genetics, management condition, DNA isolation, and sequencing strategies, make it difficult to draw a definitive conclusion from the current literature. It remains unknown whether different classes of AGPs such as classical antibiotics and ionophores modulate the intestinal microbiota in similar or distinct manners.

In this study, we directly compared the effects of five AGPs including bacitracin methylene disalicylate (BMD), tylosin, virginiamycin, monensin, and salinomycin on the cecal microbiota of broilers. These five AGPs were chosen because they are commonly used in the U.S. poultry industry and are known to have different antimicrobial spectra and mechanisms. BMD is a broad-spectrum cyclic peptide antibiotic that functions through inhibition of bacterial cell wall synthesis, while tylosin, a macrolide, and virginiamycin, a streptogramin, both target Gram-positive bacteria by inhibiting bacterial protein synthesis [2,17]. Monensin and salinomycin, on the other hand, are polyether ionophores that act against coccidia and Gram-positive bacteria by transporting ions and dissipating ion gradients across bacterial cell membranes [2,17]. Based on deep sequencing of the V3-V4 region of the bacterial 16S rRNA gene after 2-week subtherapeutic supplementation of five AGPs, we revealed in the current study an obvious shift in the structure of the cecal bacterial community, with two ionophores having the most striking effect. Identification of a number of bacterial taxa that are commonly and uniquely altered in response to different AGPs sheds new light on their growth-promoting mechanism and may allow targeted manipulation of the intestinal microbiota to improve animal health and productivity in the future.

## 2. Materials and Methods

### 2.1. Animal Trial

All animal trials were conducted in accordance with the Institutional Animal Care and Use Committee of Oklahoma State University (protocol number AG173, approved January 27, 2016). A total of 576 day-of-hatch male Cobb broiler chicks were obtained from the Cobb-Vantress Hatchery (Siloam Springs, AR, USA.) and randomly assigned to one of six dietary treatments with eight birds per cage and 12 cages per treatment in a completely randomized block design. Only Marek’s disease vaccine was given at the hatchery and no other vaccinations were provided during the trial. Upon arrival, animals received either a non-medicated standard corn-soybean starter diet formulated to meet or exceed NRC requirements or the starter diet supplemented with one of five AGPs for 14 days. The supplemental levels were BMD (0.5 g BMD^®^-50/kg diet, equivalent to 55 mg BMD/kg, Zoetis, Parsippany, NJ, USA), tylosin (0.5 g Tylan^®^-40/kg, equivalent to 44 mg tylosin/kg, Elanco Animal Health, Greenfield, IN, USA), virginiamycin (0.5 g Stafac^®^-20/kg, equivalent to 22 mg virginiamycin/kg, Phibro Animal Health, Teaneck, NJ, USA), monensin (0.5 g Coban^®^-90/kg, equivalent to 99 mg monensin/kg, Elanco Animal Health), and salinomycin (0.5 g Bio-Cox^®^-60/kg, equivalent to 66 mg salinomycin/kg, Zoetis), respectively. All antimicrobial doses are recommended at subtherapeutic levels for disease prevention or growth promotion by respective manufacturers.

The chickens were raised on floor cages with fresh pine wood shavings under standard management. Water and feed were provided ad libitum for the entire duration of the trial. The room temperature was started at 33 °C and decreased 3 °C every 7 days. The light to dark ratio was 24:0 for day 0, 23:1 for days 1 to 3, 18:6 for days 4 to 6, and 16:8 for days 7 to 14. The temperature and lighting programs were designed in accordance with Cobb-Vantress’ recommendations to ensure optimal growth. On day 14, two chicks were randomly selected from each cage and euthanized via CO_2_ asphyxiation. Cecal content was collected aseptically from each bird for microbiome analysis. The samples were immediately frozen in liquid nitrogen and stored at −80 °C until further processing.

### 2.2. DNA Extraction and Sequencing

Bacterial DNA was isolated from cecal contents using the ZR Fecal DNA Isolation Kit (Zymo Research, Irvine, CA, USA) according to the manufacturer’s protocol. The quality and quantity of DNA samples were determined using a Nanodrop ND-1000, and agarose gel electrophoresis was used to confirm the absence of degradation. High quality DNA was sequenced for the V3-V4 region of the 16S rRNA gene by Novogene (Beijing, China) on Illumina HiSeq2000 using 341F (CTAYGGGRBGCASCAG) and 806R (GGACTACNNGGGTATCTAAT) primers. Novogene’s standard protocol using the NEB Next^®^ Ultra™ Library Prep Kit was used for PCR amplification and library preparation.

### 2.3. Bioinformatic Analysis and Statistics

Raw sequences were processed using mothur, version 1.39.5 [18], according to the standard operating procedures. Low quality sequences and singletons were removed. Sequences were aligned using the SILVA database prior to classification using the RDP 16S rRNA training set 16. Sequences that shared no less than 97% identity were clustered into one operational taxonomic unit (OTU) and relative abundance was calculated. Differences in the microbial community structure were calculated using R version 3.5.1 [19]. The α- and β-diversities were calculated with the phyloseq package, version 1.24.2 [20], while plots were made using ggplot2 version 3.0.0 [21]. Statistical differences in α-diversity and relative abundance were determined using one-way ANOVA with post hoc Tukey’s test in R. The α-diversity was calculated using the Shannon evenness index and observed OTUs as measures of evenness and richness, respectively.

The β-diversity was calculated using Bray-Curtis and Jaccard indices and statistical difference in the microbiome composition was determined using analysis of similarity (ANOSIM) in the vegan package of R, version 2.5-2 [22]. Metastats [23] was used to determine significant differences in the relative abundance of each OTU between individual treatments and the control group. Venn diagrams were drawn using the VennDiagram package of R [24], and a heatmap was generated using Heatmapper [25].

### 2.4. Accession Number

Sequencing data for this experiment was deposited into NCBI SRA and can be found under the accession number PRJNA552082.

## 3. Results

### 3.1. Effect of In-Feed Antimicrobials on the Cecal Bacterial Diversity

Male Cobb broiler chicks were fed a non-medicated corn-soybean basal diet supplemented with or without one of five commonly used AGPs at subtherapeutic levels for two weeks before collection of 12 cecal content samples for each treatment. Following bacterial DNA isolation and sequencing of the V3-V4 region of the 16S rRNA gene, a total of 7,767,847 raw sequence reads were obtained with an average of 107,866 ± 11,583 sequences per sample. After quality trimming and processing, 6,522,487 reads remained and were further clustered into 2416 OTUs. Sequences were subsampled to a depth of 56,629 sequences per sample for subsequent analysis.

**Figure 1:**
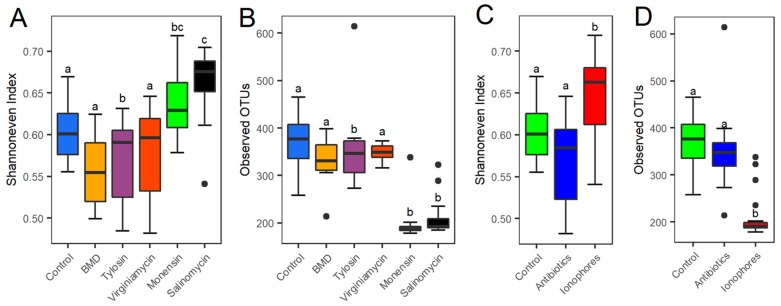
The α-diversity of cecal microbiota of broilers following 2-week supplementation of different antimicrobials. Changes in evenness and richness were calculated from 12 samples of each treatment using the Shannon evenness index (**A**) and observed operational taxonomic units (OTUs) (**B**), respectively. Data from three antibiotics and two ionophore groups were further combined separately and the Shannon evenness index (**C**) and observed OTUs (**D**) were recalculated. Results were plotted using box and whisker plots, in which the middle line denoted the median value and the lower and upper hinges represented the first and third quartiles, respectively. Whiskers extended from the hinge to the highest or lowest value no farther than 1.5 × the inter-quartile range. Points outside of this range are considered outliers. One-way ANOVA with post hoc Tukey’s test was performed, with the treatments not sharing a common superscript considered significantly different (*p* < 0.05).

The α-diversity was first calculated using Shannon evenness index (Figure 1A) and observed OTUs (Figure 1B). Both measurements revealed a trend toward a decrease in both evenness and richness of the cecal microbiota in response to three antibiotics (BMD, tylosin, and virginiamycin). Surprisingly, two ionophores (monensin and salinomycin) led to a significant increase (*p* < 0.05) in evenness of the microbiota, which was accompanied by a drastic decrease in richness (*p* < 0.05). To further reveal the difference in the cecal microbiota α-diversity between antibiotics and ionophores, data from three antibiotics and two ionophore groups were combined separately and α-diversity was calculated. Similar to individual treatments, ionophores caused a significant increase in evenness (*p* < 0.05) (Figure 1C) and a concomitant decrease in richness (*p* < 0.05) (Figure 1D), whereas the effect of antibiotics on cecal bacterial α-diversity was insignificant, suggesting that ionophores have a more pronounced effect than antibiotics on reshaping the cecal microbiota.

**Figure 2:**
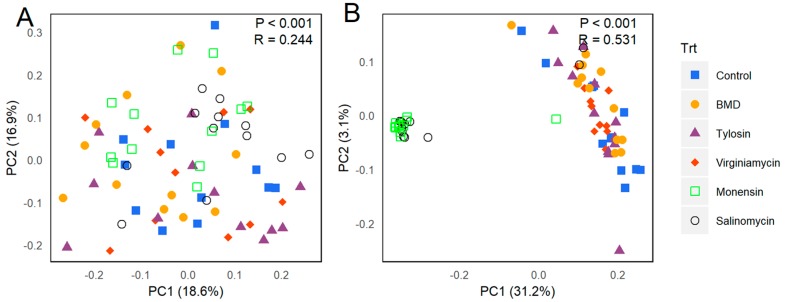
The β-diversity of cecal microbiota of broilers following 2-week supplementation of different antimicrobials. Principal coordinate analysis (PCoA) plots were generated from 12 samples of each treatment using Bray–Curtis (**A**) and Jaccard indices (**B**), respectively. Statistical significance and R values were determined using analysis of similarity (ANOSIM) and indicated in each plot.

To further reveal the differences in cecal microbiota composition among individual AGPs, β-diversity was determined using the Bray–Curtis and Jaccard indices. While there was no obvious segregation of the microbiota based on the Bray–Curtis index (Figure 2A), two ionophore groups were clearly separated from all other treatments using the Jaccard index (Figure 2B), reinforcing an earlier observation on α-diversity that in-feed ionophores had a stronger effect on cecal microbiota than antibiotics. Consistently, ANOSIM indicated that the differences in the Bray-Curtis index among treatments are mostly significant (*p* < 0.05), but generally minor (with a low R value ranging from 0.048 to 0.411) (Table 1). However, for the Jaccard index, all three antibiotics and the control group had very low R values of less than 0.1 among each other, whereas the highest R values were observed when comparing the two ionophore groups to any other group (*R* > 0.7 for all comparisons) (Table 1), in agreement with the α-diversity analysis in that two ionophores significantly reduced richness of the cecal microbiota, while three antibiotics had a relatively mild effect (Figure 1D).

### 3.2. Effect of In-Feed Antimicrobials on Cecal Bacterial Composition

**Figure 3:**
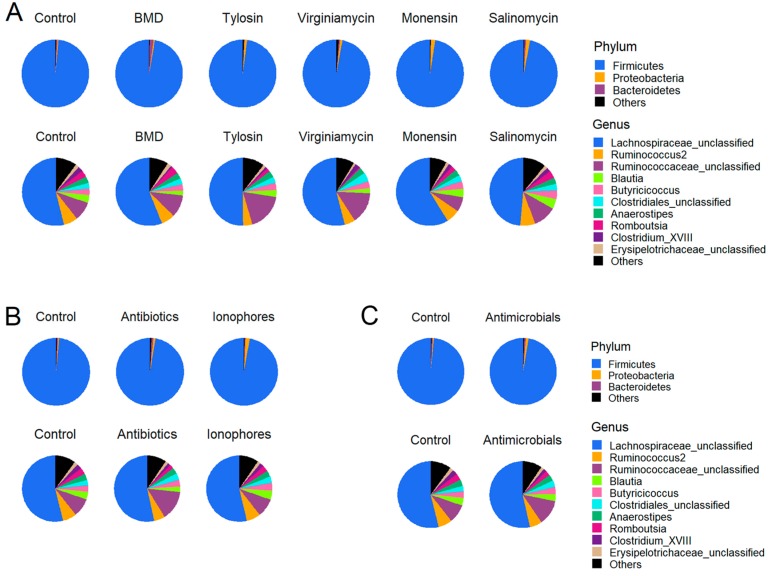
Differences in cecal microbiota composition of broilers following 2-week supplementation of different antimicrobials. Relative abundance of OTUs were calculated and plotted at the phylum and genus levels (**A**). Three antibiotics and two ionophore groups were further combined separately and relative abundance of OTUs were recalculated and plotted at the phylum and genus levels (**B**). All antimicrobial groups were combined and compared with the control group at the phylum and genus levels (**C**). Only the top three phyla and top 10 genera are shown, with unidentified and lowly abundant bacteria being collectively denoted as ‘Others’.

At the phylum level, Firmicutes was found to be the most abundant phylum accounting for over 97% of all sequences, followed by Proteobacteria and Bacteroidetes in the cecum of day-14 broilers (Figure 3A). Statistical analysis revealed no significant difference in relative abundance of Firmicutes or Proteobacteria among treatments (Supplementary Table S1). Relative abundance of Bacteroidetes and Actinobacteria varied significantly among the treatments (*p* < 0.05). Virginiamycin resulted in a significant decrease in Actinobacteria, relative to the control group, while BMD significantly increased Bacteroidetes as compared to tylosin and monensin (*p* < 0.05) (Supplementary Table S1). At the genus level, over 48% of sequences were identified as unclassifiable members of *Lachnospiraceae* (Figure 3A). Statistical analysis of the top 10 genera revealed differential effects of AGP supplementation on members of *Ruminococcaceae, Clostridiales,* and *Romboutsia*. For example, tylosin supplementation caused a significant increase in an unclassified genus of the *Ruminococcaceae* family relative to the control group (*p* < 0.05), while virginiamycin resulted in a significant diminishment of *Romboutsia* of the *Peptostreptococcaceae* family and a concomitant increase in an unclassified genus of the Clostridia class as compared to control (*p* < 0.05) (Supplementary Table S1).

When three antibiotics and two ionophore groups were combined and compared with the control group, no significant difference in relative abundance of Firmicutes, Proteobacteria, or Bacteroidetes was observed at the phylum level, while Actinobacteria was significantly reduced by antibiotics, but not ionophores (Figure 3B and Supplementary Table S2). At the genus level, a significant increase in unclassified *Ruminococcaceae* was observed in the antibiotics group over control (*p* < 0.05), while ionophores had a minimum impact (Figure 3B and Supplementary Table S2). *Romboutsia* was significantly augmented in response to antibiotics (*p* < 0.05), but remained unaltered by ionophores (Supplementary Table S2). When all five AGP groups were pooled and compared to the control (Figure 3C), Firmicutes was slightly, but significantly decreased by AGPs, while the opposite was true with Proteobacteria (Supplementary Table S3). A significant increase in an unclassified member of both *Ruminococcaceae* and *Clostridiales* was observed (*p* < 0.05), while all other genera remained largely unchanged (Supplementary Table S3).

### 3.3. Differential Regulation of OTUs by Antimicrobial Supplementation

**Figure 4:**
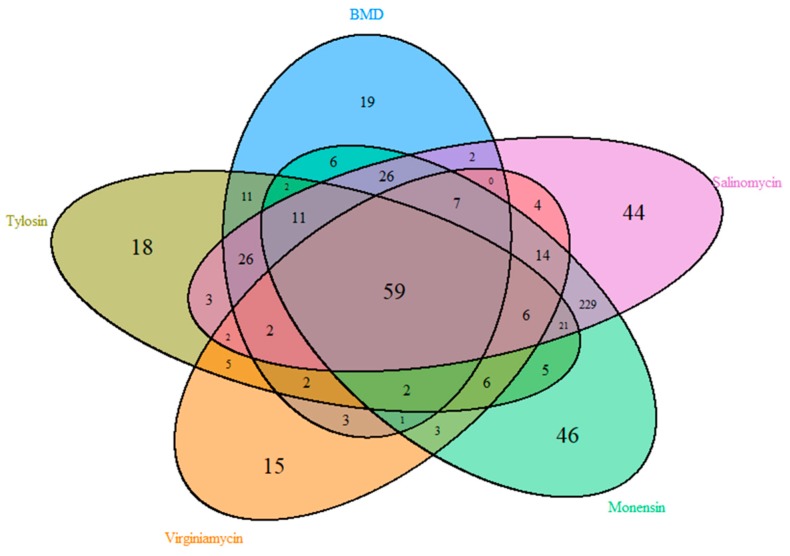
Differential enrichment of OTUs by in-feed antimicrobials. Significant up- or down-regulation of OTUs was determined using Metastats [23], relative to the control group (*p* ≤ 0.05). Venn diagram was then used to visualize the distribution of shared OTUs among individual antimicrobials.

In order to better define changes in individual OTUs, Metastats [23] was used to identify OTUs that were significantly altered (*p* ≤ 0.05) by each AGP relative to the control group. Overall, 898 OTUs were significantly up- or down-regulated by at least one AGP, with 59 of those affected by all five AGPs (Figure 4). The majority of these were observed to be rare OTUs belonging to a diverse set of bacterial genera (data not shown). Apparently, each AGP also showed an obvious differential effect, with a group of OTUs being uniquely modulated by individual AGPs (Figure 4). Additionally, monensin and salinomycin supplementation resulted in a depletion of a number of lowly abundant OTUs belonging to unclassified genera of *Ruminococcaceae* and *Clostridiales* (data not shown), consistent with a significant decrease in microbiota richness in the α-diversity analysis.

**Figure 5:**
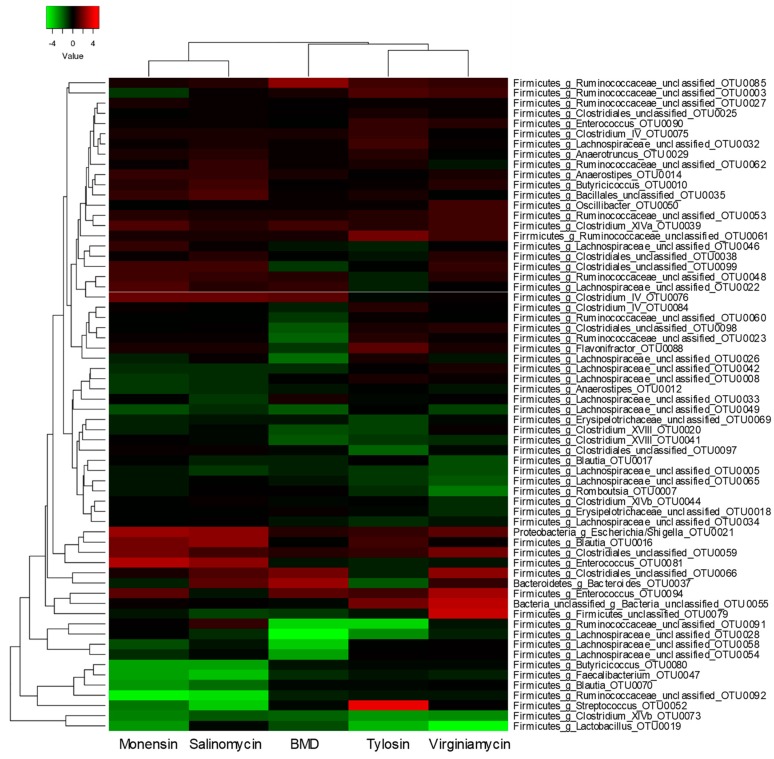
Differential regulation of the top 100 OTUs by in-feed antimicrobials. Among the top 100 OTUs, 64 were significantly affected by at least one antimicrobial and thus plotted using Heatmapper [25]. Fold change was calculated as the mean relative abundance of an OTU in an antimicrobial group relative to that in the control, followed by log_2_ transformation for visualization. Both rows and columns were clustered using the Euclidean distance and average linkage.

**Figure 6:**
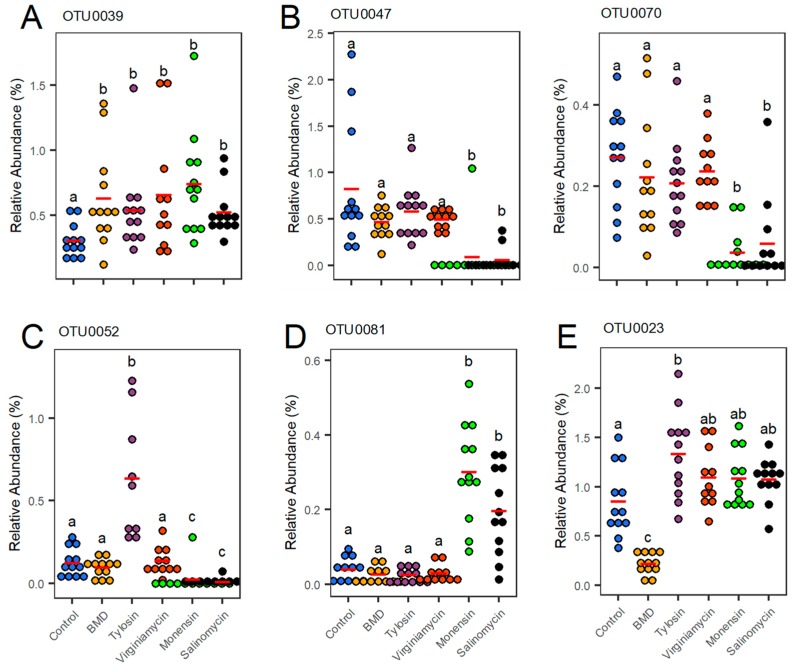
Differential regulation of representative bacteria taxa by in-feed antimicrobials. Each group consists of 12 cecal samples as indicated by each dot. The mean relative abundance of each group was indicated as a red dash. For OTU0052, three outliers (relative abundance > 3%) were omitted for better visualization of final differences among groups. Statistical significance was determined using Metastats [23], with the treatments not sharing a common letter considered significantly different (*p* < 0.05).

Among the top 100 most abundant OTUs, 64 were significantly affected by at least one treatment as revealed by Metastats and visualized in a heatmap (Figure 5). It is obvious that each AGP regulates different bacterial populations in the cecum. For example, an unclassified member of *Ruminococcaceae* (OTU0085) was significantly increased by BMD, but remained largely unchanged in response to other AGPs; on the other hand, another unclassified *Ruminococcaceae* (OTU0003) was significantly suppressed by monensin, but not other AGPs (Figure 5). However, several OTUs were regulated similarly in response to all five AGPs. For example, an *Escherichia/Shigella* member (OTU0021), a *Blautia* member (OTU0016), a *Clostridium* XlVa member (OTU0039), and an unclassified member of *Clostridiales* (OTU0059) were enriched by all five AGPs, whereas a *Clostridium* XIVb member (OTU0073) and a *Lactobacillus* (OTU0019) were decreased by all AGPs (Figure 5). Relative abundances of OTU0039 in the cecum of individual broilers were further illustrated in a dot plot (Figure 6A).

It is not surprising that Euclidean clustering identified a clear segregation of five AGPs, with three antibiotics forming one clade and two ionophores forming the other (Figure 5). A cluster of four members of Firmicutes (OTU0047, OTU0070, OTU0080, and OTU0092) was clearly suppressed by two ionophores, but largely unaffected by three antibiotics (Figure 5 and Figure 6B). A *Streptococcus* member (OTU0052) was also significantly suppressed by two ionophores, but dramatically enriched by tylosin, while two other antibiotics had no effect (Figure 5 and Figure 6C). Conversely, an *Enterococcus* (OTU0081) was significantly enriched by two ionophores, but unaffected by antibiotics (Figure 6D). Within the antibiotics clade, tylosin and virginiamycin were shown to cluster separately from BMD, consistent with their differences in antimicrobial mechanism and spectrum [2,17]. A cluster of six OTUs (OTU0023, OTU0026, OTU0060, OTU0084, OTU0088, and OTU0098) and another cluster of two OTUs (OTU0054 and OTU0058) were uniquely suppressed by BMD, with no obvious effect by other antibiotics (Figure 5) as exemplified by OTU0023 (Figure 6E).

## 4. Discussion

In-feed AGPs are known to modulate gut microbiota. Early culture-independent studies using molecular techniques such as terminal restriction fragment length polymorphism, denaturing gradient gel electrophoresis, and Sanger sequencing of the 16S rRNA gene clone libraries revealed a consistent and obvious antibiotic-induced shift in the microbiota composition [26,27,28,29,30,31]. However, these techniques lack the depth and precision to reveal specific changes in bacterial taxa. Subsequent next-generation sequencing of the bacterial 16S rRNA gene demonstrated specific changes in certain bacterial populations, but with largely inconsistent results. Multiple studies have shown no obvious effect of antibiotics on α-diversity [32,33,34,35], while others showed a decrease [36,37] or an increase in α-diversity of the cecal microbiota [38]. Significant changes in bacterial composition, measured by β-diversity, were observed more consistently [34,37,38,39], with only a few studies not reporting a shift [36,40]. However, very few studies compared the impact of multiple AGPs, particularly antibiotics and ionophores, on the gut microbiota side-by-side.

In this study, two-week supplementation with three classical antibiotics (BMD, tylosin, and virginiamycin) had a minimum effect on α- or β-diversity of the chicken cecal microbiota. Surprisingly, a significant decrease in richness and a concurrent increase in evenness of the cecal microbiota was observed for two ionophores (monensin and salinomycin), consistent with the 16S rRNA gene sequencing results showing a significant diminishment of a large number of rare bacterial phylotypes. Previous work investigating the effects of ionophores on poultry microbiota is limited. However, Danzeisen et al. [32] reported a similar, though non-significant, decrease in α-diversity of the cecal microbiota in broilers in response to monensin supplementation.

Overall, the most dramatic effect of AGP supplementation is differential enrichment of the *Clostridiales* order, particularly the members of three most dominant bacterial families in the chicken cecum (*Ruminococcaceae*, *Lachnospiraceae,* and *Clostridiaceae*) [41]. Among the 100 most abundant bacterial taxa, a number of the *Ruminococcaceae* members were enriched in the cecum by AGPs, while many *Lachnospiraceae* species appear to be diminished (Figure 5). Previous studies have found both *Ruminococcaceae* and *Lachnospiraceae* to be increased following supplementation of broiler diets with a mixture of chlortetracycline, virginiamycin, and amoxicillin [42], but decreased by avilamycin, flavophospholipol, or zinc bacitracin [36,43]. Both *Ruminococcaceae* and *Lachnospiraceae* are known to produce butyrate [44,45]. It is noted that several *Clostridium* IV and XIVa members such as OTU0039, OTU0075, and OTU0076 were also significantly increased in abundance. Both IV and XIVa clusters of Clostridia are the two most dominant bacterial taxa in the hind gut of humans and well-known for their ability to produce butyrate from indigestible carbohydrates [46,47]. These results are consistent with earlier observations that in-feed antibiotics preferentially enriched butyrate-producing bacteria [42].

Lactic acid bacteria are widely used as probiotics to provide a myriad of beneficial effects to the host [48,49]. Only three lactic acid bacterial genera including *Enterococcus, Lactobacillus*, and *Streptococcus* were differentially regulated by AGPs among the 100 most abundant OTUs in the cecum. It is interesting to note that all three *Enterococcus* members (OTU0081, OTU0090, and OTU0094) were upregulated by AGPs, while the only *Lactobacillus* member (OTU0019) was obviously reduced in response to all but one AGP. A reduction in the *Lactobacillus* abundance is consistent with earlier observations that AGP administration was associated with depopulation of the *Lactobacillus* species [50,51], resulting in reduced bile salt deconjugation and improved fat digestion and utilization [51]. Lactobacilli are known to be major producers of bile salt hydrolase responsible for hydrolyzing and deconjugating primary bile acids [52]. A lone *Streptococcus* member (OTU0052) was significantly reduced by two ionophores, but significantly enriched by tylosin, while BMD and virginiamycin had a minimum impact. *Streptococcus* has been shown to be suppressed by carbadox and a mixture of three antibiotics (chlortetracycline, sulfamethazine, and penicillin) in pigs [53] or a mixture of three different antibiotics (amoxicillin, metronidazole, and bismuth) in mice [54].

These results collectively suggest that AGPs have a strong tendency to enrich butyrate- and lactic acid-producing bacteria, while reducing bile salt hydrolase-producing bacteria, in the gastrointestinal tract. A combination of these effects could potentially lead to enhanced metabolism and utilization of dietary carbohydrates and lipids and improved energy harvest and mucosal immune defense, which may be collectively responsible for the growth-promoting effect of AGPs, although they are yet to be experimentally verified.

Among five AGPs selected for this study, BMD kills a broad spectrum of Gram-positive bacteria by interfering with synthesis of bacterial cell wall and peptidoglycan, while tylosin and virginiamycin act against a narrower spectrum of Gram-positive bacteria via inhibition of bacterial protein synthesis [17]. Two ionophores (monensin and salinomycin), on the other hand, kill bacteria and parasites by facilitating transportation of monovalent ions and thereby disrupting ion gradients across cell membranes, although monensin preferentially transports Na^+^ and H^+^, while salinomycin prefers K^+^ and Na^+^ [17,55]. An obvious differential effect exists among individual AGPs and among different classes of AGPs, although a number of bacteria are commonly regulated by all AGPs. It is apparent that the larger the difference in the antibacterial spectrum and mode of action among AGPs, the more distinct the bacterial populations that they regulate. We observed that monensin and salinomycin modulate similar bacterial populations that are rather different from three antibiotics. Among three antibiotics, virginiamycin and tylosin were found to manipulate the microbiota composition in a more similar manner than BMD.

Among the top 100 OTUs, 97 belong to Firmicutes. The only *Bacteroides* (OTU0037) was obviously enriched by BMD and salinomycin, but suppressed by tylosin and monensin. Such a differential regulation pattern is interesting, but the reason is currently unknown, although *Bacteroides,* with the ability to degrade non-digestible carbohydrates to produce short-chain fatty acids [56,57], was reported to be enriched by BMD [38]. A lone Proteobacteria (*Escherichia/Shigella* OTU0021) was also enriched by all five AGPs, which is in agreement of earlier reports of a transient upregulation of *Escherichia/Shigella* in response to in-feed antibiotics [53,54].

It is important to note that closely related bacteria within a genus are not necessarily regulated in the same fashion even by the same AGP. For example, multiple clusters of the *Clostridium* genus were differentially regulated. One abundant member of *Butyricicoccus* (OTU0010) was enriched by AGPs, but a less abundant *Butyricicoccus* (OTU0080) was diminished particularly by ionophores. While *Blautia* OTU0016 is upregulated, *Blautia* OTU0017 and OTU0070 are downregulated by AGPs. It is, therefore, difficult to predict the net outcome in the abundance of certain bacterial populations. Future studies to perform absolute quantification of individual bacterial taxa will help provide a more definitive answer to the net change in each bacterial population.

In this study, we demonstrated the differential effects of five different AGPs on the cecal microbiome of broiler chickens. While each treatment displayed certain effect on cecal microbiome composition, the most drastic changes were observed with ionophores. Investigation into the regulation of specific OTUs revealed an enrichment of beneficial bacteria following antimicrobial treatment, particularly in OTUs involved in butyrate synthesis. However, we only examined the cecal microbiota changes two weeks after AGP supplementation. It will be beneficial to investigate the kinetic response of the gut microbiota in response to AGPs, revealing whether certain bacterial populations undergo temporal or persistent alterations. Furthermore, because the small intestine is the major site where most nutrients are digested and absorbed, studying the microbiota changes in the small intestine by AGPs is also warranted. Additionally, we only focused on luminal microbiota in this study. Mucosa-associated microbiota is also intimately associated with host metabolism and immune response and it would be of great interest to study its alteration in response to different AGPs in the future.

Although different AGPs appear to shift the structure of gut microbiota in distinct manners, it will be important to examine how the function of gut microbiota is altered by AGPs, which can be evaluated by using a combination of metagenomics, metabolomics, metatranscriptomics, and/or metaproteomics. It is tempting to speculate that, regardless of the antimicrobial spectrum and mode of action, AGPs improve growth performance by enhancing the functional potential of the gut microbiota resulting in more efficient digestion and utilization of dietary carbohydrates and lipids in the gastrointestinal tract.

In summary, our data indicates an ability of AGPs to modulate intestinal microbiota to allow an increase in the bacteria associated with improved digestion and energy utilization. A better understanding of the mechanism by which AGPs modulate gut microbiota and enhance growth of livestock animals will lead to the development of effective antibiotic alternatives that mimic the action of AGPs.

## Figures and Tables

**Table 1:** Pairwise comparisons of different antimicrobials on β-diversity of the cecal microbiota using the ANOSIM analysis.

	Control	BMD	Tylosin	Virginiamycin	Monensin	Salinomycyin
**Control**		*p* = 0.043 *R* = 0.071	*p =* 0.052 *R* = 0.061	*p =* 0.036 *R* = 0.090	*p* < 0.001 *R* = 0.921	*p* < 0.001 *R* = 0.809
**BMD**	*p =* 0.013 ^1^ *R* = 0.145		*p =* 0.243 *R* = 0.021	*p =* 0.043 *R* = 0.095	*p* < 0.001 *R* = 0.914	*p* < 0.001 *R* = 0.774
**Tylosin**	*p =* 0.009 *R* = 0.149	*p =* 0.004 *R* = 0.187		*p =* 0.595 *R* = 0.015	*p* < 0.001 *R* = 0.855	*p* < 0.001 *R* = 0.714
**Virginiamycin**	*p* = 0.001 *R* = 0.226	*p =* 0.009 *R* = 0.164	*p =* 0.197 *R* = 0.048		*p* < 0.001 *R* = 0.957	*p* < 0.001 *R* = 0.832
**Monensin**	*p* < 0.001 *R* = 0.315	*p* < 0.001 *R* = 0.284	*p* < 0.001 *R* = 0.411	*p* < 0.001 *R* = 0.401		*p =* 0.021 *R* = 0.076
**Salinomycin**	*p =* 0.002 *R* = 0.163	*p =* 0.003 *R* = 0.205	*p* < 0.001 *R* = 0.294	*p* < 0.001 *R* = 0.292	*p =* 0.029 *R* = 0.105	

^1^ Shaded *p*- and R-values are for the Bray-Curtis index, while non-shaded *p*- and R-values represent the Jaccard index.

## Supplementary Materials

The following are available online at https://www.mdpi.com/2076-2607/7/9/282/s1.
