# Supplementary material for: Differential Impact of Subtherapeutic Antibiotics and Ionophores on Intestinal Microbiota of Broilers
Source: Microorganisms. 2019 Aug 22;7(9):282. doi: 10.3390/microorganisms7090282 (PMC6780560; doi:10.3390/microorganisms7090282)
Supplement: Supplementary file 1 [file microorganisms-07-00282-s001.pdf]

**Supplementary Table 1. Effect of in-feed antimicrobials on the cecal bacteria composition**

| Taxonomy | Control | BMD | Tylosin | Virginiamycin | Monensin | Salinomycin | *P*_Value |
|---|---|---|---|---|---|---|---|
| **Phylum** | | | | | | | |
| Firmicutes | 98.5 | 97.2 | 97.9 | 97.2 | 97.6 | 97.0 | 0.692 |
| Proteobacteria | 0.44 | 0.60 | 1.16 | 1.02 | 1.93 | 1.87 | 0.468 |
| Bacteroidetes | $0.61^{ab}$ | $1.85^{a}$ | $0.27^{b}$ | $0.65^{ab}$ | $0.23^{b}$ | $0.87^{ab}$ | 0.016 |
| Actinobacteria | $0.15^{a}$ | $0.10^{ab}$ | $0.08^{ab}$ | $0.07^{b}$ | $0.11^{ab}$ | $0.13^{ab}$ | 0.019 |
| Others | $0.24^{b}$ | $0.20^{b}$ | $0.54^{ab}$ | $1.07^{a}$ | $0.15^{b}$ | $0.12^{b}$ | <0.001 |
| **Genus** | | | | | | | |
| *Lachnospiraceae*_unclassified | 53.91 | 56.11 | 50.17 | 54.06 | 58.89 | 48.56 | 0.135 |
| *Ruminococcaceae*_unclassified | $9.11^{bc}$ | $10.63^{bc}$ | $17.96^{a}$ | $15.09^{ab}$ | $7.00^{c}$ | $11.17^{abc}$ | <0.001 |
| *Ruminococcus2* | 6.74 | 6.52 | 4.41 | 5.13 | 6.53 | 7.13 | 0.585 |
| *Butyricicoccus* | 3.15 | 3.10 | 3.53 | 3.46 | 3.77 | 4.14 | 0.101 |
| *Blautia* | 3.66 | 2.25 | 3.27 | 2.38 | 3.98 | 4.66 | 0.082 |
| *Clostridiales*_unclassified | $2.69^{b}$ | $2.89^{b}$ | $2.89^{b}$ | $4.29^{a}$ | $2.98^{ab}$ | $3.19^{ab}$ | 0.014 |
| *Anaerostipes* | 3.17 | 2.93 | 3.17 | 2.97 | 3.000 | 2.99 | 0.997 |
| *Romboutsia* | $3.25^{ac}$ | $3.65^{c}$ | $1.75^{ab}$ | $0.65^{b}$ | $2.44^{ac}$ | $3.67^{c}$ | <0.001 |
| *Clostridium*_XVIII | 2.42 | 0.81 | 1.02 | 2.27 | 1.79 | 1.99 | 0.091 |
| *Erysipelotrichaceae*_unclassified | 1.80 | 1.99 | 1.40 | 1.05 | 1.49 | 1.58 | 0.294 |

Note: Mean relative abundance (%) of the bacterial phylum and top 10 genera are shown, with 12 samples per treatment. Statistical significance was determined using one-way ANOVA and *post hoc* Tukey's test. Columns not sharing a common superscript are considered significantly different ($p < 0.05$).

**Supplementary Table 2. Differential regulation of the cecal bacterial composition by antibiotics and ionophores.**

| Taxonomy | Control | Antibiotics | Ionophores | *P*-Value |
|---|---|---|---|---|
| **Phylum** | | | | |
| Firmicutes | 98.54 | 97.45 | 97.29 | 0.346 |
| Proteobacteria | 0.44 | 0.92 | 1.9 | 0.119 |
| Bacteroidetes | 0.61 | 0.93 | 0.55 | 0.478 |
| Actinobacteria | $0.15^{a}$ | $0.09^{b}$ | $0.12^{ab}$ | 0.003 |
| Others | $0.24^{a}$ | $0.59^{a}$ | $0.13^{b}$ | 0.016 |
| **Genera** | | | | |
| *Lachnospiraceae*_unclassified | 53.91 | 53.43 | 53.73 | 0.988 |
| *Ruminococcaceae*_unclassified | $9.11^{a}$ | $14.54^{b}$ | $9.10^{a}$ | 0.002 |
| *Ruminococcus2* | 6.74 | 5.36 | 6.83 | 0.350 |
| *Butyricicoccus* | 3.15 | 3.36 | 3.96 | 0.028 |
| *Blautia* | $3.66^{ab}$ | $2.64^{a}$ | $4.32^{b}$ | 0.019 |
| *Clostridiales*_unclassified | 2.69 | 3.33 | 3.09 | 0.267 |
| *Anaerostipes* | 3.17 | 3.03 | 2.99 | 0.938 |
| *Romboutsia* | $3.25^{ab}$ | $2.05^{a}$ | $3.06^{b}$ | 0.019 |
| *Clostridium*_XVIII | 2.42 | 1.34 | 1.89 | 0.125 |
| *Erysipelotrichaceae*_unclassified | 1.80 | 1.49 | 1.53 | 0.644 |

Note: Mean relative abundance (%) of the bacterial phylum and top 10 genera are shown, with 12 samples per treatment. Statistical significance was determined using one-way ANOVA and *post hoc* Tukey's test. Columns not sharing a common superscript are considered significantly different ($p < 0.05$).

**Supplementary Table 3. Regulation of the cecal bacterial composition by antimicrobials**

| **Taxonomy** | **Control** | **Antimicrobials** | ***P*-Value** |
|---|---|---|---|
| **Phylum** | | | |
| Firmicutes | 98.54[a] | 97.39[b] | 0.013 |
| Proteobacteria | 0.44[a] | 1.32[b] | 0.008 |
| Bacteroidetes | 0.61 | 0.78 | 0.581 |
| Actinobacteria | 0.15 | 0.10 | 0.088 |
| Others | 0.24 | 0.41 | 0.115 |
| **Genera** | | | |
| *Lachnospiraceae*_unclassified | 53.91 | 53.55 | 0.887 |
| *Ruminococcaceae*_unclassified | 9.11[a] | 12.32[b] | 0.009 |
| *Ruminococcus2* | 6.74 | 5.95 | 0.568 |
| *Butyricicoccus* | 3.15 | 3.60 | 0.268 |
| *Blautia* | 3.66 | 3.32 | 0.591 |
| *Clostridiales*_unclassified | 2.69[a] | 3.23[b] | 0.037 |
| *Anaerostipes* | 3.17 | 3.01 | 0.771 |
| *Romboutsia* | 3.25 | 2.46 | 0.189 |
| *Clostridium*_XVIII | 2.42 | 1.56 | 0.206 |
| *Erysipelotrichaceae*_unclassified | 1.80 | 1.51 | 0.319 |

Note: Mean relative abundance (%) of the bacterial phylum and top 10 genera are shown, with 12 samples per treatment. Statistical significance was determined using Student's t-test. Columns not sharing a common superscript are considered significantly different ($p < 0.05$).
